# Tension band wiring for simple olecranon fractures: evaluation of surgical technique

**DOI:** 10.1007/s10195-017-0450-2

**Published:** 2017-02-28

**Authors:** Femke M. A. P. Claessen, Michel P. J. van den Bekerom, C. Niek van Dijk, J. Carel Goslings, Gino M. M. J. Kerkhoffs, Job N. Doornberg, On behalf of the Shoulder elbow platform, E. Hoebink, P. Nolte, P. Eggen, R. Blokzijl, D. Haverkamp, D. L. van Deurzen, A. Vochteloo, T. Kraal, A. Peters, E. E. J. Raven, M. M. Campo, A. Heijink, E. Mutsaerts, S. A. F. Tulner, J. R. Lansdaal, J. Jenner, C. C. J. Jaspars, R. A. van den Wijngaard, A. J. M. Janus, E. M. Nelissen, M. van der Pluijm, H. van der Bracht, R. van Hove, D. Broekhuis, M. P. Somford, P. J. Damen, P. Scholten, J. J. Wiegerinck, C. Schonhuth, D. van Kampen, D. Eygendaal, B. van Ooij, L. Plaat, T. G. Guitton, S. B. Schouten, J. Hermans, E. W. Zwitser, R. J. P. van der Wal, G. A. Buijze, A. van Tongel, D. van Oostveen, S. van de Velde

**Affiliations:** 1 Shoulder and Elbow Unit, Onze Lieve Vrouwe Gasthuis, Amsterdam, Netherlands; 2 Department of Orthopaedic Surgery, Academic Medical Center Amsterdam, Amsterdam, The Netherlands; 3 Trauma Unit Department of Surgery, Academic Medical Center Amsterdam, Amsterdam, The Netherlands; 4 Orthopaedic Sports and Traumatology, Department of Orthopaedic Surgery, Academic Medical Center Amsterdam, Amsterdam, The Netherlands; 5 Orthotrauma Research Center, University of Amsterdam Orthopaedic Residency Program (PGY 4), Amsterdam, The Netherlands; 6 Orthopaedic Hand and Upper Extremity Service, Yawkey Center, Massachusetts General Hospital, Harvard Medical School and University of Amsterdam Medical School, 55 Fruit Street, Boston, MA 02114 USA

**Keywords:** Elbow trauma, Olecranon fracture, Tension band, Technique pain, Disability

## Abstract

**Background:**

In some settings, specific techniques for open reduction and internal fixation are preferred based on the eminence of a surgeon or professional organization. An emphasis on technical aspects of surgery that are not proved superior and vary substantially from surgeon to surgeon can be confusing for trainees. This study applied a numerical grading of the technical aspects of tension band wire (TBW) fixation for olecranon fracture; assessed the interobserver agreement of each criterion; and measured the correlation of the technical grading and objective and subjective long-term outcomes.

**Materials and methods:**

Forty observers were invited to rate the technical aspects of TBW fixation of the olecranon on 26 post-operative radiographs. The interobserver reliability of the rating was measured using the intra-class correlation coefficient. The correlation between the rating and motion, Mayo elbow performance index, and disabilities of the arm, shoulder and hand score was tested with the Spearman’s rank correlation test.

**Results:**

None of the figure-of-eight TBW constructs were considered perfect according to the numerical grading: the majority of observers found three deviations per fixation. The interobserver agreement was only fair for the total number of deviations and no correlation between the number of deviations and long-term objective and subjective outcome was found.

**Conclusions:**

A rating of the technical aspects of TBW for olecranon fractures was unreliable and did not correlate with subjective and objective outcomes. Emphasis on specific technical aspects of fixation might be confusing for trainees and could distract them from the principles of effective treatment.

**Level of evidence:**

Level IV diagnostic study.

## Introduction

Operative fixation is indicated for most olecranon fractures-especially displaced olecranon fractures in healthy, active patients [15]. Operative treatment of displaced, transverse, non-comminuted fracture of the olecranon is associated with good to excellent elbow function in retrospective short-term follow-up studies [2, 14]. Moreover, satisfactory clinical results are durable over time [6, 10].

The tension band principle as applied to transverse olecranon fractures fixed by tension band wiring (TBW) is based on the premise that distraction forces on the outer cortex of the ulna during elbow flexion are converted to compression forces on the articular surface of the olecranon at the fracture site [14]. The specific technical aspects of the TBW for simple olecranon fracture are subject to ongoing debate as there is little evidence to support any specific technique [5, 9, 13, 14]. Nevertheless, the specific surgical technique or technical aspects of the procedure are preferred based more upon the eminence of the surgeon or group rather than the data. For instance, the AO technique emphasizes parallel and intramedullary Kirschner wires, but it is not clear that those specific technical aspects confer an advantage [5, 9, 13, 14].

Surgeons mostly agree with themselves (good to excellent intra-observer reliability), but not so much with each other (poor interobserver reliability) (Claessen et al. unpublished data). An emphasis on technical aspects of surgery that are not proved superior and vary substantially from surgeon to surgeon can be confusing and demoralizing for trainees. For example, some surgeons prefer intramedullary Kirschner wires and others transcortical fixation. A discussion of the variations is instructive, but criticism for not following one surgeon’s preferences sends the wrong message.

Schneider et al. [14] described ten criteria for evaluating surgical treatment of olecranon fractures based on ten operative imperfections: (1) nonparallel K-wires, (2) long K-wires, (3) K-wires extending radially outwards, (4) insufficient fixation of the proximal ends of the K-wires, (5) intramedullary K-wires, (6) perforation of the joint surface, (7) single wire knot, (8) jutting wire knot(s), (9) loose figure-of-eight configuration, and (10) incorrect repositioning to evaluate radiographs of olecranon fractures.

This study applied a numerical grading of the technical aspects of TBW fixation for olecranon fracture based on the Schneider criteria [14]; assessed the interobserver agreement of the rating; and measured the correlation of the technical grading and objective and subjective long-term outcomes. We hypothesized that the average number of observed technical deviations according to the Schneider criteria per TBW will be at least three. Our secondary hypothesis is that interobserver agreement on the total number of technical deviations is poor. We also hypothesized that there is no correlation between technical deviations of the TBW surgical technique and long-term objective and subjective clinical outcome.

## Materials and methods

### Study design

Our local Medical Ethics Committee approved this interobserver study to evaluate 26 postoperative-anonymized radiographs of patients treated for olecranon fractures at the Academic Medical Center between 1974 and 1997, with subjective and objective outcome scores available after 10–30 years follow-up [6]. From 1974, all trauma patients treated and admitted to our level I trauma center were prospectively documented in a trauma database classified according to the arbeitsgemeinschaft für osteosynthesefragen (AO) comprehensive classification of fractures.

We previously reported on the long-term subjective (DASH) and objective outcomes (Broberg and Morrey elbow arthritis score, range of motion) of 41 patients [6].

Inclusion criteria were: (1) traumatic non-pathological simple olecranon fracture, and (2) age 18 years or older. Patients were excluded if no post-operative lateral and anterior–posterior radiograph was available.

Of those 41 patients, 26 patients had a traumatic non-pathological simple olecranon fracture and a post-operative lateral and anterior–posterior radiograph.

All included patients underwent open reduction internal fixation (ORIF) for transverse noncomminuted olecranon fractures between 1977 and 1997. During this time period, our general indication for performing ORIF was greater than 2 mm displacement. In this study, indication for TBW was a simple transverse noncomminuted olecranon fracture.

The average age of the included patients was 34 years (range: 19–72 years). Nine patients were female (35%) and 17 were male (65%). The average follow-up time was 18 years (range 9–33). The average flexion extension arc was 139 degrees (range 95–150). The average postoperative DASH score was nine (range 0–65). Eight of 26 patients had a DASH score of greater than ten points (13; 14; 17; 17; 25; 30; 37; 65 points, respectively). The average elbow arthritis score according to Broberg and Morrey was 0.2 (range, grade 0–1). All patients started with passive range of motion exercises within 1 week.

Members of the Shoulderelbowplatform, an online collaboration of shoulder and elbow surgeons from all over the world, were invited to evaluate the radiographs on a web-based study platform including DICOM viewer. Members of the Shoulderelbowplatform are fully trained, actively practicing surgeons and residents from around the world. The goals of the Shoulderelbowplatform are to: (1) facilitate online interobserver reliability and diagnostic accuracy studies about orthopedic shoulder and elbow injuries, (2) offer a platform to educate residents.

### Participants

Sixty-three senior orthopaedic residents and orthopaedic surgeons started to evaluate 26 radiographs on the platform (www.shoulderelbowplatform.com), of which 40 observers finished the study (63% of the initial responders). Twenty-one orthopaedic residents and 19 orthopaedic surgeons completed the study. Thirty-five percent of the observers were less than 3 years in practice and the majority of the observers were involved in resident training (53%) (Table 1).

**Table 1:** Observer demographics (*n* = 40)

Demographic	Number (%)
Sex	
Male	38 (95)
Female	2 (5)
Years in practice	
<3	14 (35)
4–6	11 (27.5)
7–10	8 (20)
11–20	6 (15)
21–30	1 (2.5)
Involvement in resident training	
Yes	21 (52.5)
No	19 (47.5)
Number of olecranon fractures treated per year	
<5	23 (57.5)
6–10	13 (32.5)
11–20	4 (10)
>20	0 (0)

### Study description

After login, postoperative radiographs (standard anterior–posterior and lateral views) of 26 treated olecranon fractures were presented to the observers. Observers were asked to critique the following details of applied surgical technique of the “classic” TBW construct on 26 post-operative radiographs based on the written description in the recent paper by Schneider et al. that we would like to coin the Schneider criteria [14] (Table 2). In total 10 points per TBW construct could be obtained. A point was given for each of the 10 Schneider criteria that did not meet expectations.

**Table 2:** Schneider criteria

Schneider criteria
Oversized Kirschner wires in terms of length
Loose figure-of-eight configuration (i.e. the wire cerclage not ‘flush’ to the bone)
Incorrect reduction (i.e. congruent joint articular surface)
Perforation of the joint surface
Non-parallel Kirschner wires (with reference to the other Kirschner wire) on anterior–posterior view
Kirschner wires extending radially outwards
Proximal ends of the Kirschner wires not bent 180 degrees back into the cortical bone of the olecranon
Two intramedullary Kirschner wires
Single wire knot
Prominent wire knot(s) (i.e. twisted ends not sufficiently bent back into direct contact with the bone)

**Fig. 1:**
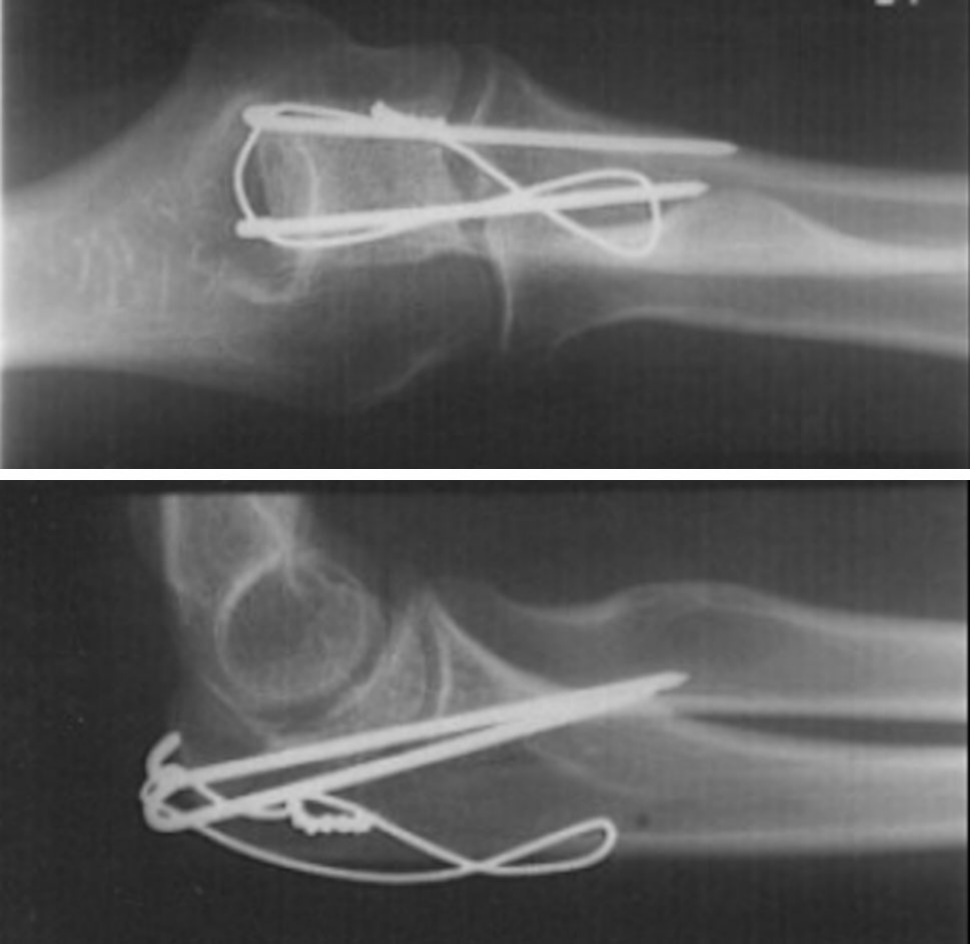
Antero–posterior and lateral radiograph of a simple olecranon fracture. It can be conflicting if twisted ends of the wire knot were sufficiently bent back into direct contact with the bone

**Fig. 2:**
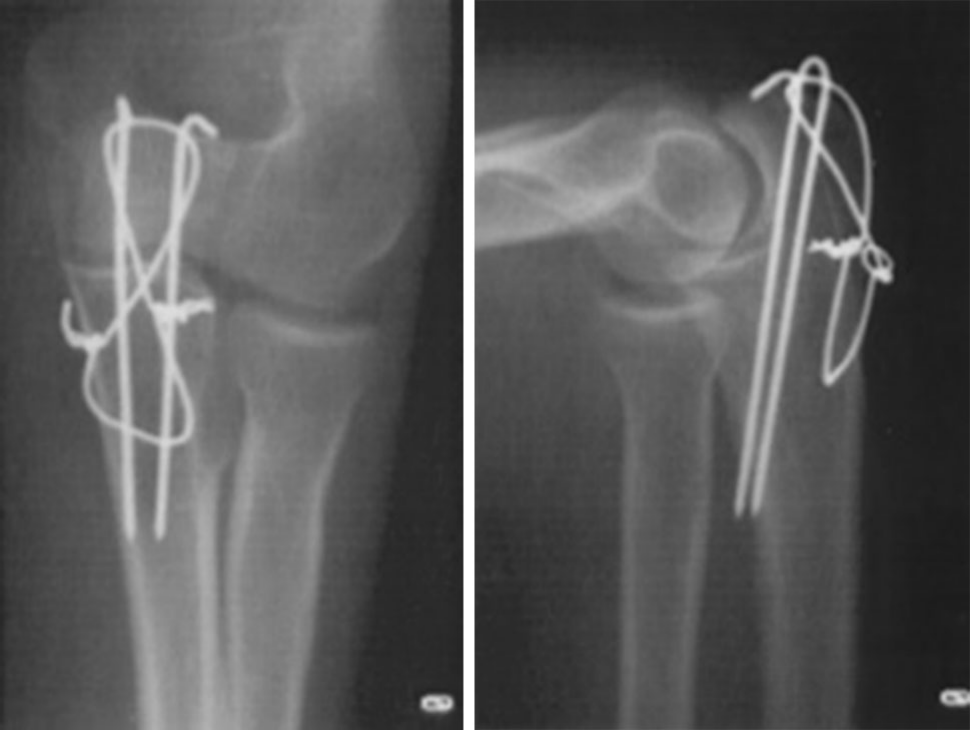
Antero–posterior and lateral radiograph of a simple olecranon fracture. It can be conflicting if the Kirschner wires were oversized in term of length and if twisted ends of the wire knot were sufficiently bent back into direct contact with the bone

The following Schneider’s criteria for evaluating TBW of olecranon fractures were subject of interpretation: oversized Kirschner wires in terms of length, loose figure-of-eight configuration (i.e. the wire cerclage not ‘flush’ to the bone), incorrect reduction (i.e. congruent joint articular surface), prominent wire knot(s) (i.e. twisted ends not sufficiently bent back into direct contact with the bone) (Figs. 1, 2).

Observers were also asked if they (1) would have performed the fixation differently and (2) advised a revision surgery.

One case had to be completed to be able to continue with the next case. The observers completed the study at their own pace and in their own time on various computers if necessary.

### Post hoc power analysis

Post hoc power analysis, performed with the use of nQuery Advisor software, revealed that 17 fractures evaluated by 34 observers would provide 80% power ($\alpha$ = 0.05, $\beta$ = 0.20) to detect a clinically significant difference [8].

### Explanatory and outcome measures

The outcome measures were the number of technical deviations according to the Schneider criteria, the interobserver agreement and long-term subjective (DASH) and objective outcomes (Broberg and Morrey elbow arthritis score, range of motion). Technical deviation was defined as a presumed technical error as proposed by Schneider et al. [14]. However, no clinical data exist that the listed technical deviations are indeed errors or mistakes, as we these have not been associated with worse clinical outcome in clinical series to date.

The explanatory variables were TBW technique characteristics.

### Statistical analysis

We assessed an intra-class correlation coefficient (ICC) two-way mixed-effects model. ICC is a measure of agreement between observers, adjusted for agreement due to chance alone used for comparison of continuous numeric data. Potential ICC values ranging from 0 (no agreement) to 1 (perfect agreement). ICC values representing poor agreement are 0.00–0.20; fair agreement, 0.21–0.40; moderate agreement, 0.41–0.60; substantial agreement, 0.61–0.80; and almost perfect agreement, greater than 0.80 [3, 12].

The Spearman’s rank correlation coefficient was done to correlate the number of technical deviations based on consensus agreement (>50% of observers) to the DASH score, the range of motion and the Broberg and Morrey elbow arthritis score.

## Results

### Numerical grading

The average number of observed technical deviations on the guideline per TBW construct was 3.0 (range 1.5–4.7) and no fixation was considered perfect (Table 3). Moreover, the observers recommended performing 96% of the fixations differently (based on the question would you have performed the fixation differently).

**Table 3:** Average number of flaws per case

Case	Average number deviations	% of observers that would perform the surgery differently	% of observers that recommend revision	F–E Arc	E/F	Mayo elbow performance index	Arthrosis score	DASH score
21	4.7	97.5	70	135	5–140	100	0	1
24	4.1	87.5	60	140	0–140	100	0	0
4	3.7	85	35	135	5–140	100	1	14
20	3.7	95	20	140	0–140	100	0	1
1	3.5	90	0	145	0–145	100	0	1
5	3.5	70	8	95	15–110	65	0	37
9	3.4	85	2.5	145	5–150	100	0	17
22	3.4	87.5	57.5	150	0–150	100	0	2
16	3.3	80	5	135	10–145	100	1	0
26	3.3	90	22.5	100	35–135	95	1	3
2	3.2	83	23	145	10–155	100	0	65
3	3.1	60	10	155	0–155	100	0	1
18	3.2	82.5	20	135	5–140	100	0	13
12	3.0	87.5	2.5	155	0–155	100	0	0
17	2.9	85	0	140	0–140	100	0	25
19	2.9	67.5	40	130	0–130	85	0	17
11	2.8	92.5	8	130	0–130	100	0	1
13	2.7	87.5	5	150	5–155	100	0	0
6	2.6	85	15	155	5–150	100	0	0
23	2.6	95	27.5	160	10–150	100	0	0
14	2.4	87.5	0	140	0–140	100	0	0
25	2.3	82.5	5	130	5–135	100	0	3
10	1.9	87.5	0	140	0–140	100	0	1
15	1.9	87.5	8	125	5–130	100	1	0
8	1.8	22.5	8	150	5–155	45	0	30
7	1.5	57.5	8	150	–150	100	0	8
Total	3.0	81.5	17.7 Average	139		96	0.2	9

*F* flexion, *E* extension, *DASH* disabilities of the arm, shoulder and hand

In almost all 26 patients, at least one observer identified one of the respective technical deviations. In other words, all potential deviations on the guideline were seen by at least one surgeon in each patient; even conflicting potential technical deviations like scoring both intramedullary Kirschner wires and Kirschner wires protruding radially in the same TBW construct by different surgeons exemplifies the fact that surgeons mostly agree with themselves (good to excellent intra-observer reliability), but not so much with each other (poor interobserver reliability) in most studies [4, 7, 16].

Only one patient had implant failure and reoperation (patient 20). There were four potential technical deviations in this TBW construct according to a consensus agreement of more than 50% of the observers: (1) non-parallel Kirschner wires (2) too long Kirschner wires (3) insufficient fixation of the proximal ends of the Kirschner wires (4) intramedullary Kirschner wires. However, the majority of surgeons in this study advised no revision of internal fixation for this patient.

### Interobserver agreement

The interobserver agreement on the total number of deviations was fair (ICC = 0.32) (reference value 0.21–0.40) (Table 4). The interobserver agreement was fair for all subgroups. For example, the interobserver agreement was not higher if the observer had more than 6 years of experience compared to less than 6 years of experience. This included a consecutive series of ratings by experts in the field of elbow surgery.

**Table 4:** Interobserver agreement total number of deficiencies

Criterium	Categorical	Intra class correlation coefficient (CI)
Total number of deviations per case	Fair agreement	0.32 (0.21–0.48)

*CI* confidence interval

### Correlation number of potential technical deviations TBW and long-term objective- and subjective outcomes

There was no correlation between technical deviations of the TBW surgical technique and long-term objective and subjective outcomes [6]. No correlation between the DASH score and the number of technical deviations was seen (*p* = 0.64). There was also no correlation between the elbow arthritis score according to Broberg and Morrey and the number of technical deviations (*p* = 0.99) [1]. However, elbow arthritis was correlated to a higher DASH score in this patient group *p* = 0.02). With the numbers available, there was no significant correlation between the number of technical deviations and range of motion: flexion (*p* = 0.06), extension (*p* = 0.07), supination (*p* = 0.23), and pronation (*p* = 0.76).

## Discussion

There is little data to support one technique over the other for TBW of olecranon fractures [5, 9, 13, 14]. In this study we applied a numerical grading of the technical aspects of TBW fixation for olecranon fracture; assessed the interobserver agreement of each criterion; and measured the correlation of the technical grading and objective and subjective long-term outcomes.

The average figure-of-eight TBW construct of a displaced-transverse-non-comminuted olecranon fracture in this series had at least three out of ten potential technical deviations according to a consensus agreement of greater than 50% of 40 observers [14]. In other words, pearls and pitfalls of TBW technique for simple olecranon fractures will remain a subject of ongoing debate, which can be confusing to the trainee. The point of discussion should be the lack of consensus and the dearth of evidence, not the preferences of any given surgeon. Schneider et al. evaluated 233 TBW constructs for ten potential technical deviations—the Schneider criteria [14]. They found an average of 4.24 imperfections per TBW construct and concluded that TBW is not as easy as surgeons and published reports suggest [14].

We would have expected higher than fair agreement on the total number of technical deviations per TBW construct given that some potential technical deviations were not subject to interpretation, such as single wire knot and prominent wire knot(s).

The lack of correlation between technical deviations and outcomes suggest that most of the Schneider criteria [14] are irrelevant from the patient’s perspective. The range of motion of the elbow in our study is also discording from the number of surgeons who suggested a revision surgery (in several patients an inferior ROM corresponded to a better judgment on radiographs, and vice versa). Perhaps olecranon fractures have a wide margin for error and a good outcome is likely except for technical mistakes and severe non-compliance. The evidence that nonoperative treatment and olecranon nonunion lead to good function in most patients supports this idea [2, 14]. In any case, the clinical outcome cannot be predicted based on postoperative radiographs.

This study should be interpreted in light of several limitations. First of all, this interobserver reliability lacks a reference standard, as the configuration of a “perfect” TBW construct is unknown and therefore accuracy data could not be calculated. The radiographs were all made according to hospital protocol, but were not otherwise standardized. However, this represents our daily clinical practice. A high proportion of the observers were residents and young surgeons. However, the interobserver agreement was fair for all subgroups. Observers had no information regarding patient characteristics or injury. Also, we did not give observers any training or reference values on technique specifics, only the written description as above. Perforation of the surface joint might be underestimated on plain films and the gold standard here in case of doubt might be computed tomography in any future reference study. Due to low number of implant loosening or breakage (one patient, 4%) we lack power to correlate any potential technical deviations to loss of fixation. According to a post hoc power analysis a 25% complication rate was needed to detect any correlation between technical deviations and loss of fixation.

A numerical grading of technical aspects of the TBW fixation for olecranon fracture was unreliable and did not correlate with objective and subjective long-term outcomes. In other words, technic specifics of the TBW fixation will remain a subject of ongoing debate, which might be confusing to the trainee and might distract them from the principles of effective treatment. The discussion should be focused on the lack of consensus and the minimal evidence, not the preferences of the surgeon.
