# Correction to: Tension band wiring for simple olecranon fractures: evaluation of surgical technique

**DOI:** 10.1186/s10195-018-0512-0

**Published:** 2018-09-06

**Authors:** Femke M. A. P. Claessen, Michel P. J. van den Bekerom, C. Niek van Dijk, J. Carel Goslings, Gino M. M. J. Kerkhoffs, Job N. Doornberg, the Shoulder Elbow Platform, E. Hoebink, P. Nolte, P. Eggen, R. Blokzijl, D. Haverkamp, D. L. van Deurzen, A. Vochteloo, T. Kraal, A. Peters, E. E. J. Raven, M. M. Campo, A. Heijink, E. Mutsaerts, S. A. F. Tulner, J. R. Lansdaal, J. Jenner, C. C. J. Jaspars, R. A. van den Wijngaard, A. J. M. Janus, E. M. Nelissen, M. van der Pluijm, H. van der Bracht, R. van Hove, D. Broekhuis, M. P. Somford, P. J. Damen, P. Scholten, J. J. Wiegerinck, C. Schonhuth, D. van Kampen, D. Eygendaal, B. van Ooij, L. Plaat, T. G. Guitton, S. B. Schouten, J. Hermans, E. W. Zwitser, R. J. P. van der Wal, G. A. Buijze, A. van Tongel, D. van Oostveen, S. van de Velde

**Affiliations:** 1 Shoulder and Elbow Unit, Onze Lieve Vrouwe Gasthuis, Amsterdam, Netherlands; 2 Department of Orthopaedic Surgery, Academic Medical Center Amsterdam, Amsterdam, The Netherlands; 3 Trauma Unit Department of Surgery, Academic Medical Center Amsterdam, Amsterdam, The Netherlands; 4 Orthopaedic Sports and Traumatology, Department of Orthopaedic Surgery, Academic Medical Center Amsterdam, Amsterdam, The Netherlands; 5 Orthotrauma Research Center, University of Amsterdam Orthopaedic Residency Program (PGY 4), Amsterdam, The Netherlands; 6 Orthopaedic Hand and Upper Extremity Service, Yawkey Center, Massachusetts General Hospital, Harvard Medical School and University of Amsterdam Medical School, 55 Fruit Street, Boston, MA 02114 USA

## Correction to: J Orthop Traumatol (2017) 18:275–281 http://doi.org/10.1007/s10195-017-0450-2

Unfortunately, after publication of this article [1], it was noticed that the author J. Carel Goslings was tagged incorrectly during the production process. This resulted in the PubMed display of the author name as ‘J Carel Goslings’. The correct display is ‘Goslings JC’. This correction contains the correct tagging.

## References

[1] Claessen FMAP, van den Bekerom MPJ, van Dijk CN, Goslings JC, Kerkhoffs GMMJ, Doornberg JN, On behalf of the Shoulder Elbow Platform (2017). Tension band wiring for simple olecranon fractures: evaluation of surgical technique. J Orthop Traumatol.

